# High-Throughput Screening in Protein Engineering: Recent Advances and Future Perspectives

**DOI:** 10.3390/ijms161024918

**Published:** 2015-10-20

**Authors:** Magdalena Wójcik, Aline Telzerow, Wim J. Quax, Ykelien L. Boersma

**Affiliations:** Groningen Research Institute of Pharmacy, Department of Pharmaceutical Biology, University of Groningen, A. Deusinglaan 1, 9717 AV Groningen, The Netherlands; E-Mails: m.wojcik@rug.nl (M.W.); a.telzerow@student.rug.nl (A.T.)

**Keywords:** high-throughput screening, FACS-based screening platforms, microfluidics-based screening platforms, protein engineering, *in vitro* compartmentalization

## Abstract

Over the last three decades, protein engineering has established itself as an important tool for the development of enzymes and (therapeutic) proteins with improved characteristics. New mutagenesis techniques and computational design tools have greatly aided in the advancement of protein engineering. Yet, one of the pivotal components to further advance protein engineering strategies is the high-throughput screening of variants. Compartmentalization is one of the key features allowing miniaturization and acceleration of screening. This review focuses on novel screening technologies applied in protein engineering, highlighting flow cytometry- and microfluidics-based platforms.

## 1. Introduction

Nature offers many different biocatalysts and (therapeutic) proteins, which usually require alterations in order to meet criteria with regard to reaction conditions and usage under industrial or medical circumstances. Protein engineering techniques can aid in the evolution of properties such as activity, stability, expression, selectivity and inhibition [1,2]. In the last few decades, much progress has been made in this field due to advances in mutagenesis including directed evolution, semi-rational approaches and computational methods. Nowadays, structural and mechanistic knowledge of the protein of interest as well as bioinformatics tools are applied in the prediction of hot spots [2]; consequently, small focused libraries are created which can be screened in a short time [3]. Thus, significant improvements in protein characteristics can be achieved. However, finding the best approach to generate a mutant library is only the starting point in the roadmap to a molecule with the optimally desired characteristics [4]. The choice of a suitable screening or selection system is one of the most important checkpoints in the design of an experiment [4,5]. In general, selection strategies are less labor-intensive and more efficient than screening techniques: whole libraries of variants can be analyzed simultaneously. Thus, the size of the library—usually 10^10^–10^13^ variants—is not a limiting factor. In screening strategies, each variant’s activity is determined individually; the size of the library is therefore usually much smaller, though the use of robotics has made the screening process less labor-intensive [6]. Despite the clear difference in concept and in library size that can be tested, the words screening and selection appear to be interchangeable in current literature.

The choice for a screening strategy is dictated by some constraints: a reliable production host and its transformation efficiency, assay development (compounds selection, detection limits) and the availability of analytical tools and standardized equipment [4]. All currently available strategies, such as agar plate screens, microtiter plate screens, cell surface display selections or screens of single (bacterial) cells functioning as microreactors, have their advantages and disadvantages [7]. In search for a higher throughput, compartmentalization techniques have emerged. Inspired by nature where reactions are taking place within cells or organelles, water-in-oil droplets of the size of *Escherichia coli* bacteria have been developed. These cell-like chambers brought some new advantages to screening, one of the main benefits being the miniaturization of the reaction volume to a few microliters. As a consequence, a higher throughput can be achieved, which means screening a higher number of variants in a shorter amount of time [8,9]. However, reducing reaction volumes has some limitations: the reduction is associated with physical restrictions in terms of evaporation and capillarity [10]. Thus, a further decrease in reaction volume was impossible until the introduction of microfluidics technology, which enabled the decrease of the reaction volume down to a few picoliters [9,11].

Two techniques have emerged in the field of protein engineering together with the development of compartments: fluorescence-activated cell sorting (FACS) and microfluidics. This review focuses on advances in the application of these methods in the context of protein engineering, with an emphasis on applications in enzyme engineering. Chosen for their novelty and general applicability, recent examples from the last five years are described here to illustrate the use of high-throughput screening technologies; these examples are summarized in Table 1 and Table 2.

## 2. Nature’s Own Compartments in FACS-Based Screening Platforms

A prerequisite of any screening or selection method is a physical link between the genotype and the resulting phenotype [5]; this is typically a ‘compartment’, such as prokaryotic or eukaryotic cells. Thus, the impact of the change in the nucleic acid sequence can be observed and characterized at the level of the expression product, the protein [8]. Several screening and selection strategies based on nature’s own compartments have been developed, e.g., mammalian cell display, yeast surface display and cell-based screening; these have been combined with FACS to achieve a higher throughput of screening [6,7]. Evolution of the protein of interest must lead to a fluorescent signal for sorting. Fluorescent (non-)natural probes are commercially available, although natural substrates should preferably be used to prevent false positive results: e.g., the protein of interest could strongly bind to the non-natural fluorescent probe or not show any improvement in activity towards its natural substrate [12].

### 2.1. Display of Protein Variants on Eukaryotic Cells

Antibody display systems using phages, yeast cells or ribosomes have seen many successes in the development of new therapeutics. However, only small antibody fragments can be displayed; reformatting of these fragments into soluble, full-length IgGs is not always an easy task. In addition, the selected antibody fragments may possess either different posttranslational modifications or none at all [13,14,15]. To isolate and affinity mature antibodies, several mammalian display methods have been developed, which usually rely on transient transfection of mammalian cells, resulting in rapid protein expression. Episomal vectors can be stably transfected at high efficiency and maintained at low copy number; hence, multiple rounds of selection are now possible [16]. Bowers *et al.* describe a generalized approach that imitates the adaptive immune system by coupling *in vitro* somatic hypermutation (SHM) with mammalian cell display [14]. In the adaptive immune system, SHM is used to mature antibodies and generate high-affinity antibody responses through the enzymatic activity of activation-induced cytidine deaminase (AID). This enzyme targets DNA encoding the immunoglobulin variable domains, and preferentially deaminates cytidine residues at hotspot motifs [17,18].

In non-B cells, SHM can be achieved by expression of recombinant AID. HEK293 cells were stably transfected with episomal vectors expressing a library based on germline V-gene segments with recombined human regions. A C-terminal transmembrane domain was added to the full-length IgG1 heavy chain (HC) to anchor the antibody on the cell surface. Antibodies were selected in two rounds against the target antigen human β-neuronal growth factor (hβNGF). After sequencing analysis, six unique clones were chosen for affinity maturation via SHM. Then, an additional three rounds of sorting were performed with increasing stringency, and the best hβNGF-binding antibody-expressing cells were selected; sequencing revealed enriched mutations, in particular in the HC regions. The best mutant APE925 was characterized having a *K*_D_ of 25 pM [14].

**Figure 1:**
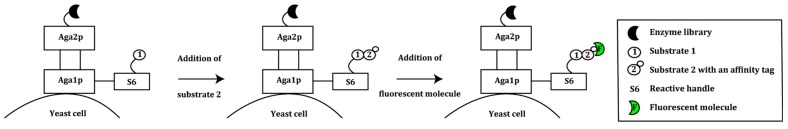
An overview of the yeast display system used in the evolution of bond-forming enzymes. The enzyme library is displayed on the yeast surface fused to the Aga2p mating factor. Aga2p is bound covalently to the Aga1p mating factor. Substrate 1 is linked to the system via the reactive handle S6. Substrate 2, which is conjugated to an affinity tag, is added to the system. Only active library members will catalyze the reaction between substrate 1 and 2. This is followed by the addition of a fluorescent molecule that binds to the affinity handle and screening using FACS [20].

Yeast display has been used frequently for the improvement of affinity [19]. It is however not often applied in directed enzyme evolution. Integrating yeast display as one of the main elements, Chen *et al.* developed a general method for the evolution of bond-forming enzymes with an improved catalytic activity [20]. In this method, an enzyme library is anchored on the yeast cell surface as a fusion to the Aga2p cell surface mating factor. The latter is covalently bound to the Aga1p mating factor via the S6 peptide which functions as a reactive handle. *Bacillus subtilis* Sfp phosphopantetheinyl transferase enables covalent attachment of substrate 1 to the S6 peptide. Substrate 2, conjugated to an affinity tag (e.g., biotin), is added to the substrate 1-conjugated enzyme library displayed on yeast cells. Following incubation with substrate 2, cells are stained with a fluorescently labeled molecule that can bind to substrate 2’s affinity tag, enabling isolation by FACS (Figure 1).

To validate the system, the transpeptidase sortase A (SrtA) from *Staphylococcus aureus* was used. SrtA recognizes an LPXTG motif (X being any amino acid) and cleaves the scissile amide bond between threonine and glycine using a nucleophilic cysteine residue. The resulting acyl-enzyme intermediate is resolved with (GGG)*_n_*-linked molecules resulting in a fusion peptide or protein [21]. Wild type SrtA was randomly mutated using an error prone PCR (epPCR) method and the resulting library was displayed on the *Saccharomyces cerevisiae* strain ICY200. The SrtA substrate GGGYK was covalently linked to the S6 peptide, while the second substrate, a biotinylated peptide conjugated to an LPETG recognition motif, was added exogenously. Incubation of the enzyme library with the substrates was followed by addition of fluorescently labeled streptavidin to detect the biotinylated LPETG peptide. In four rounds, the cells were screened with FACS under increasing selection pressure; the surviving genes were subjected to DNA shuffling, displayed on yeast cells and sorted again four times. After the final round, the extent of product formation was 40-fold higher than that of wild type SrtA. One variant with four mutations exhibited a 140-fold increase in LPETG-coupling activity (*k*_cat_/*K*_M_) compared to the wild type, though showing a decreased GGG substrate binding [20]. Further engineering of this variant led to the construction of the pentamutant eSrtA which showed a 120-fold higher *k*_cat_/*K*_M_ for the LPETG substrate and a 20-fold higher *K*_M_ for the GGG substrate.

The pentamutant eSrtA from the previous study was used as a starting point in the reprogramming of the enzyme’s substrate specificity towards the substrates L**A**ETG or LPE**S**G [22]. eSrtA has a 103-fold preference for LPETG over L**A**ETG, and a five-fold preference for LPETG over LPE**S**G. The *eSrtA* gene was subjected to chemical mutagenesis, and the library was displayed on the yeast surface, together with the L**A**ETG or LPE**S**G substrate. After three rounds of screening and further mutagenesis steps, the best variants showed a 51,000-fold change in specificity for L**A**ETG instead of LPETG, and a 125-fold change in specificity for LPE**S**G instead of LPETG. Importantly, there was no loss in catalytic efficiency compared to eSrtA [22].

**Figure 2:**
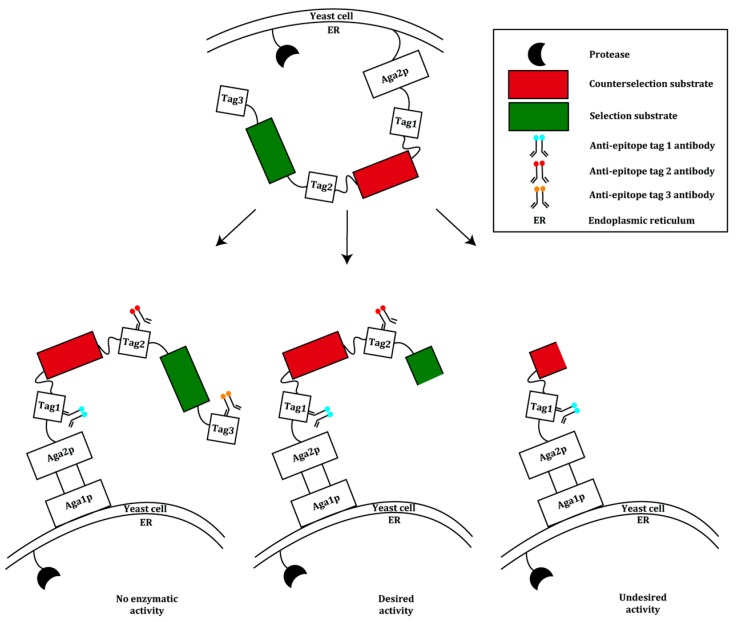
Schematic overview of the yeast endoplasmic reticulum sequestration screening (YESS) system. Both protease and substrate contain a C-terminal endoplasmic reticulum (ER) retention sequence. Within the ER, the protease can move in close proximity to the substrate. Depending on the specificity of the engineered protease, proteolysis of the selection substrate or counterselection sequence takes place. This results in the removal of the ER retention sequence and respective epitope tags located on the substrate fusion polypeptide. The remaining N-terminal portion of the polypeptide is then displayed on the yeast cell surface. Each cell is labeled with fluorescently conjugated anti-epitope tag antibodies and screened using multicolor FACS [24].

Yeast endoplasmic reticulum sequestration screening (YESS) was developed as a general, highly versatile eukaryotic system for directed evolution of proteolytic substrate selectivity and activity [23,24]. YESS is based on the co-expression of a tobacco etch virus protease (TEVp) mutant library as a fusion with multifunctional sequences. These include sequences encoding yeast adhesion receptor subunit Aga2, selection and counterselection substrates flanked by epitope tags, and an ER retention signal. Once the ER retention sequence and respective epitope tags are cleaved off by the protease, what remains of the (counter-) selection substrate will migrate to the *S. cerevisiae* cell surface; there, it will be anchored to the cell wall via Aga2, and the epitope tags can be detected by fluorescently labeled anti-epitope antibodies (Figure 2). The availability of these epitope tags depends on which substrate is cleaved. Multicolor FACS can be used to sort cells exhibiting a characteristic fluorescence profile corresponding to the selective proteolysis only at the desired substrate sequence. As a consequence, rare cells expressing a protease variant capable of specifically cleaving at only the desired new sequence are enriched. Using a counterselection substrate is key to avoiding isolating mutants with relaxed specificity.

Yi *et al.* validated YESS by altering the substrate specificity of TEVp using the *S. cerevisiae* strain EBY100 (*URA*^+^/*leu^−^*/*trp*^−^). The protease recognizes the amino acid sequence ENLYFQS/G [25] and will cleave between the glutamine (P1) and serine or glycine residues. The substrate sequence was randomized at position P1 (ENLYF**Q**S→ENLYF**X**S, X being any amino acid) to create a substrate library. The natural substrate peptide ENLYFQS was used for counterselection. Four residues at the TEVp S1 pocket were subjected to saturation mutagenesis as well. In three consecutive rounds, 3.3 × 10^7^ mutants were screened. Sequencing of 50 selected clones identified 35 different mutant TEVp-substrate combinations, with TEVp variants selectively recognizing six different amino acids at position P1. Variants PE3 and PH7, preferring a glutamate and a histidine, respectively, at position P1, were selected for further evolution of the catalytic activity. Five rounds of FACS sorting resulted in the identification of 17 clones with increased catalytic activity, with two clones, TEV-PE10 and TEV-PH21, showing the highest activity. TEV-PE10 showed a 13-fold higher *k*_cat_/*K*_M_ value for its preferred ENLYF**E**S substrate compared to the wild type substrate, resulting in 5000-fold reversal of substrate specificity. Similarly, TEV-PH21 showed a seven-fold higher *k*_cat_/*K*_M_ for the ENLYF**H**S substrate compared to the wild type substrate; this corresponds to an 1100-fold reversal of substrate specificity compared to the wild type TEVp.

To demonstrate the generality of the YESS system for the directed evolution of proteases, analogous constructs were created for hepatitis C virus protease (HCV-P) and human granzyme K (GrK) in conjunction with their preferred substrate sequences. Yeast cells displaying these constructs showed a relatively similar fluorescence readout compared to controls lacking proteases. In addition, Yi *et al.* showed that the YESS system could also be used for the detection of tyrosine phosphorylation by human Abelson tyrosine kinase (AblTK). Promising results from these experiments indicate that the YESS system could have a broader applicability in directed evolution [24].

### 2.2. Prokaryotic Cytoplasmic Screening of Protein Variants

Kostallas and Samuelson used bacterial cells to develop a cytoplasmic screening platform for the *in vivo* activity and solubility of proteases, as not all enzymes can be expressed in yeast cells. In their proof-of-principle enrichment experiments, TEVp was used [26]. In this method, *E. coli* DH5α cells are used for the constitutive expression of green fluorescent protein (GFP) fused to ssrA^NY^, and co-expression of TEVp variants. ssrA^NY^, an optimized variant of ssrA [27], functions as a degradation tag which is recognized by the cytoplasmic protease ClpXP, resulting in degradation of GFP and elimination of fluorescence [28,29]. Introduction of a protease cleavage site between the GFP and the ssrA^NY^ genes enables selection of a substrate-specific protease: the ssrA^NY^-tag will be removed and, therefore, GFP will be salvaged from degradation. The resulting fluorescence intensity correlates with protease activity and permits cell sorting with FACS [24]. Using this system, differences in the substrate processing efficiency of the wild type TEVp were analyzed. Two fusion proteins, GFP-subG-ssrA^NY^ containing the natural TEVp cleavage site ENLYFQ**G** and GFP-subP-ssrA^NY^ with the non-canonical ENLYFQ**P** sequence were used. In a proof-of-principle experiment, cells co-expressing TEVp and GFP-subG-ssrA^NY^ were mixed with cells co-expressing TEVp and GFP-subP-ssrA^NY^ in a ratio of 1:100,000. The mixture was sorted in two rounds of FACS; 71% of the most fluorescent population appeared to express the wild type substrate GFP-subG-ssrA^NY^. This is translated into a 69,000-fold enrichment of cells expressing the GFP-subG-ssrA^NY^ fusion compared to the starting point [26].

To assess TEVp’s substrate specificity, three combinatorial substrate libraries with sequences XNLXFXG, XNLXFXX and EXXYXQX (X being any amino acid) were constructed and constitutively expressed in *E. coli* DH5α. The libraries were co-expressed with TEVp and fluorescent clones were collected. After two rounds of sorting, more than 78% of the clones from all libraries showed high fluorescence intensity. Sequencing results showed that the selected peptide substrates had sequences identical to or having high sequence similarity with the wild type substrate ENLYFQGS. Furthermore, no clones expressed a better proteolytic substrate in terms of *k*_cat_/*K*_M_ than the wild type substrate ENLYFQGS. Though this method corroborates TEVp’s wild type substrate sequence, it cannot confirm the site at which cleavage actually takes place. This could be a limiting factor for proteases of which the substrate profile is not as well studied as for TEVp [30].

**Figure 3:**
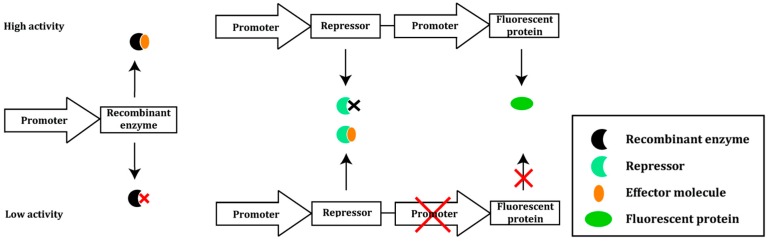
Scheme showing the ligand-mediated *eGFP*-expression system (LiMEx). Regulation of a fluorescent protein is dependent on the competitive relationship between the effector molecule and the enzymatic performance of a co-expressed recombinant enzyme. Upon high enzymatic activity, the effector is depleted (indicated with a black cross), which results in expression of the fluorescent protein. In contrast, an enzyme variant with low activity is unable to bind the effector molecule, which is subsequently intercepted by the repressor. Together with the effector molecule, the repressor binds tightly to the promoter region of the fluorescent protein and suppresses the expression of the protein (indicated with a red cross). The signal derived from the fluorescent probe is screened using flow cytometry [31].

The previously mentioned FACS-based screening platforms are based on a fluorescence signal proportional to a specific activity or affinity. Recently, a novel FACS-based screening system for the detection of subtle differences in intracellular metabolite concentrations was described. This ligand-mediated eGFP-expression system (LiMEx) establishes a competitive relationship between the regulation of a fluorescent protein by an effector molecule and the biochemical depletion of this effector molecule by a co-expressed recombinant enzyme [31]. The fluorescence intensity originating from the fluorescent protein can thus be used to quantify the enzymatic activity. Cheng *et al.* used the regulation of arginine biosynthesis in *E. coli* to validate the LiMEx system. This amino acid is synthesized from citrulline by employing argininosuccinate synthetase (ASS) and argininosuccinate lyase (ASL) [32]. ASS is encoded by *argG*, the transcription of which is regulated by the arginine repressor (ArgR). As *eGFP* is under the control of the *argG* promoter, tight binding of ArgR in the presence of co-repressor arginine to the *argG* promoter region suppresses eGFP expression. Thus, the intracellular arginine concentration, even in the micromolar range, can be correlated to eGFP fluorescence under physiological conditions (Figure 3).

The arginine concentration can be varied by the activity of a co-expressed arginine-metabolizing enzyme. To this purpose, *Pseudomonas plecoglossicida* arginine deiminase (PpADI) was co-expressed in *E. coli*; expression of the enzyme will lead to a decrease in the intracellular arginine concentration and a correlated increase in eGFP fluorescence. The previously evolved PpADI mutant M21 was used as a template for randomization by epPCR. After three rounds of directed evolution, PpADI activity was detected in 90% of the sorted clones, with 30% of that population showing at least a 1.5-fold improvement compared to the parent M21. The best variant, M31, showed a 2.8-fold increase in *k*_cat_/*K*_M_ compared to the parent M21, which corresponded to a 970-fold increase in catalytic efficiency compared to the wild type PpADI [31].

## 3. Man-Made Compartments in FACS Screening Platforms

As described above, maintaining a link between genotype and phenotype is naturally ensured by compartmentalization of the library in whole cells. Nevertheless, the use of cells significantly reduces the size of libraries that can be sampled due to the need of transformations or transfections. Furthermore, as cell viability must be maintained, the scope of buffers, solvents and temperatures that can be used are limited [33]. In the last two decades, the field of cell-free directed evolution has rapidly grown and expanded with the emergence of *in vitro* compartmentalization techniques.

### 3.1. Emulsion-Based Compartments

The first compartments were water-in-oil droplets, formed by mixing water with a mineral oil containing an adjuvant surfactant [8]. However, as these droplets are not compatible with FACS-based screening, it is necessary to cover the continuous oil phase with an external aqueous phase. The resulting water-in-oil-in-water compartments or double emulsions are compatible with flow cytometry, thus broadening the applicability of emulsion-based compartments [34].

**Figure 4:**
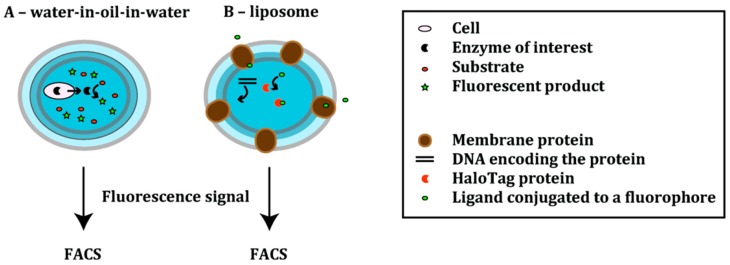
Schemes showing the emulsion-based compartments. Scheme (**A**) gives a general overview of the ViPer [12,35] and the ProFC-IVC [36] methods. Enzyme-secreting cells are encapsulated together with the substrate into double emulsions. The reaction between the secreted enzyme and the substrate results in the formation of a fluorescent product. The signal is measured using flow cytometry; Scheme (**B**) represents a novel liposome display system [37]. In this cell-free translation system, DNA encoding a membrane protein of interest is encapsulated in a cell-sized phospholipid vesicle together with a HaloTag protein. After the membrane protein’s expression and formation of pores, small molecules—ligands conjugated to a fluorophore—are able to enter the liposome and react with the HaloTag protein. The transporter activity of the membrane protein is correlated with the fluorescence signal, which is measured using flow cytometry.

Ostafe *et al.* developed an assay for the detection of cellulase-based decomposition into soluble sugars. The so-called ViPer assay (Figure 4A) is based on the indirect measurement of the reduction of sugars [12,35]. Cellulases convert cellulosic substrates into glucose monomers or polymers. These in turn are converted by hexose oxidase to produce hydrogen peroxide; the latter is used by vanadium bromoperoxidase (VBrPOx) in the conversion of aminophenyl fluorescein (APF) into fluorescein. The accumulation of fluorescein is thus a measure of the cellulase activity and can be used to sort cells [12]. Only the cellulases are expressed in *S. cerevisiae*; the cells are *in vitro* compartmentalized together with all other necessary enzymes and substrates.

In proof-of-principle experiments, this assay was used to enrich *S. cerevisiae* YPH500 cells expressing Cel5A cellulase from a mixture of 5% cellulase-positive cells and 95% cellulase-negative *S. cerevisiae* YPH500 cells transformed with the empty vector. The cells were emulsified together with all other components required for the assay in double emulsions. After one round of selection, cells expressing Cel5A showed accumulation of fluorescein in the droplets and could be sorted resulting in a 12-fold enrichment of positive cells. Although this system has only been validated in proof-of-principle experiments, it could be used to sort cellulase libraries in search of more active variants [12].

The so-called ProFC-IVC technique (Figure 4A) was developed as a screening system to evolve proteases with increased resistance to inhibitors [36]. Here, *Bacillus subtilis* WB800N cells are compartmentalized in water-in-oil-in-water droplets. As this strain is deficient in extracellular proteases, cells will only express and secrete the protease of interest while background signals are kept to a minimum [36]. Both cells and substrate, a rhodamine 110-containing peptide, are entrapped in a double emulsion in the presence of the inhibitor antipain dihydrochloride [36]. The secreted protease of interest then converts a soluble substrate into a fluorescent product; thus, the protease activity is correlated with the fluorescence intensity.

As proof of principle, the model protease subtilisin Carlsberg (SC) was subjected to epPCR. Cells expressing SC variants were encapsulated with the inhibitor antipain dihydrochloride and the peptide substrate bis(suc-AAPF)-rhodamine110; due to the negative charge of the succinyl group, the substrate was retained within the emulsion droplets. The library, containing ~10^5^ variants, was screened in three rounds of FACS to identify variants with increased resistance towards the antipain dihydrochloride inhibitor. After the final round, the library could be enriched approximately 12-fold compared to the unsorted library. A small fraction of the sorted cells was analyzed for their protease activity. One clone exhibited a 160% increase in residual activity in the presence of the inhibitor compared to the wild type, despite a reduced absolute activity [36]. This system can likely be modified to select for other properties (e.g., thermostability, altered pH profile or ionic strength resistance) or to evolve other proteases.

To extend compartmentalization techniques to the directed evolution of membrane proteins, Fujii *et al.* developed a novel liposome display system (Figure 4B) [37]. Liposome display enables evolution of membrane proteins based on their transporter activity. In a cell-free translation system, DNA encoding the membrane protein of interest and a HaloTag protein are mixed. The mixture is encapsulated in liposomes in such a way that each liposome contains a single DNA molecule. The membrane protein is expressed *in vitro* in the lipid bilayer of cell-sized, unilamellar liposome [38,39].

Proof of concept experiments were carried out using the pore-forming α-hemolysin (AH) from *S. aureus*. The AH gene was randomly mutated by epPCR and variants were selected for improved pore-forming activity. Within the liposome, AH was expressed as monomers in the aqueous phase. The water-soluble monomers spontaneously integrated into the lipid bilayer, where they associated into heptamers. Consequently, pores were formed, which enabled molecules with a molecular weight smaller than 3 kDa to enter the liposome [40]. For the detection of the pore-forming activity of α-hemolysin, a HaloTag ligand conjugated to AlexaFluor 488 (AF488) was added to the outer solution. The ligand could only cross the lipid bilayer via the AH-formed nanopores; in the liposome, the ligand covalently bound to the HaloTagged protein. Accumulation of AF488 fluorescence was therefore indicative of a greater number of pores formed and thus a higher AH activity. The top 1% population showing the highest fluorescence was sorted; after 20 rounds of selection, a mutant was identified that showed a 30-fold higher pore-forming activity compared to the wild type.

This method can potentially be applied to a variety of membrane proteins that have been integrated successfully into lipid-bilayer membranes (e.g., Fo-a subunit of F1Fo-ATP synthase [41], (1,3)-β-d-glucan (curdlan) synthase (CrdS) [42] or a multidrug transporter (emrE) [43]). Furthermore, liposome display is compatible with multimeric proteins, thus enabling the selection of homo- and heterooligomeric proteins [37].

### 3.2. Polymer-Based Platforms

**Figure 5:**
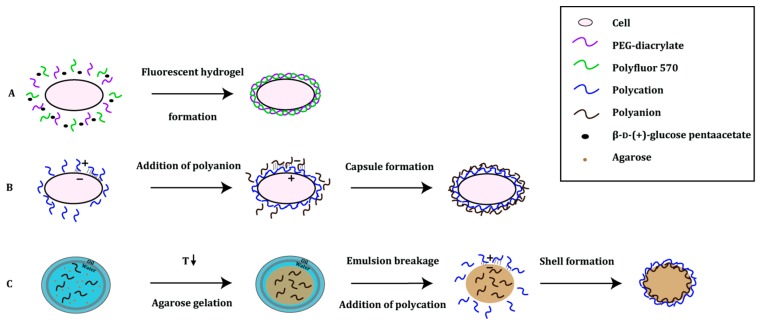
Principle of polymer-based platforms. (**A**) The fur shell method relies on the coupled reactions of two enzymes in which d-δ-gluconolactone is produced. The latter is a substrate in the Fenton reaction, which leads to the production of hydroxyl radicals, needed in the co-polymerization of poly(ethylene glycol)-diacrylate (PEG-diacrylate) and Polyfluor 570 [45,50]; (**B**) In cellular high-througput encapsulation solubilization and screening (CHESS), the polymers are assembled in a layer-by-layer manner. Each cell is subjected to the deposition of alternating layers of oppositely charged polymers [51,52]; and (**C**) Gel-shell beads are produced with the help of a microfluidic device. Water-in-oil droplets (indicated with a blue circle), containing agarose and a polyanion, are formed. With a decrease in temperature to 4 °C, agarose gelates within the water droplet (indicated with a brown circle). To decrease the molecular weight cut-off, the beads are de-emulsified in the presence of a polycation. Driven by the opposite charges, the polyanion and polycation form a shell bead that encloses any compounds of the reaction [48].

Artificial compartments can also be created using polymers. Polymer-based compartments are highly permeable and biocompatible. They are built from scratch in a stepwise manner either by means of a polyelectrolyte multilayer assembly [44] or a “self-encapsulation” radical reaction process [45] (Figure 5). In the first system, the polyelectrolyte multilayer technology takes advantage of opposing electrostatic charges of the polymers (e.g., anionic alginate, cationic chitosan). When this layer-by-layer (LbL) assembly process is finished, the innermost layer—the template of the established compartment—is removed. Remaining layers enveloping the hollow structure of the capsule function as a multilayer scaffold [44]. This approach has already been successfully used for the encapsulation of yeast and bacterial cells [46,47,48]. In the “self-encapsulation” method, the capsule is produced by means of a free radical polymerization of poly(ethylene glycol) acrylate. The polymerization reaction is triggered by hydroxyl radicals, products of a Fenton reaction between hydrogen peroxide and Fe^2+^ ions [49].

The advantage of the polymer-based compartments over water-in-oil emulsions is their high stability in aqueous solutions; this allows them to be analysed by standard FACS equipment directly. In addition, polyelectrolytes can withstand more extreme conditions than emulsion-based droplets. Finally, the semipermeable nature of the polymer-based compartments makes them suitable for multistep processes, in which the exchange of different reaction components needs to be adjustable in comparison to emulsion-based droplets [48].

The so-called fur-shell screening platform was developed as a generally applicable screening system for hydrolase activity [45]. In this method, an enzyme reaction of a glucose oxidase coupled with an active hydrolase leads to hydrogel shell formation and co-polymerization of fluorescent methacryloxyethyl thiocarbamoyl rhodamine B monomer (Polyfluor 570) around *E. coli* cells; the resulting shell resembles a fur and is fluorescent, which makes this technique compatible with FACS [45,50].

This method was used to improve the catalytic activity of *Yersinia mollaretti* phytase (YmPh) towards the non-natural substrate 4-methylumbelliferyl phosphate (4-MUP). An epPCR mutant library of YmPh was expressed in *E. coli* BL21(DE3) LacI^Q1^ cells. YmPh-catalyzed hydrolysis of glucose-6-phosphate led to the formation of β-d-glucose, which was oxidized by glucose oxidase oxidized to δ-gluconolactone under formation of hydrogen peroxide. Via the Fenton reaction, co-polymerization of the PEG-acrylate monomer with fluorescent Polyfluor 570 was triggered, thus forming a PEG-Polyfluor 570 hydrogel shell. As only cells containing active YmPh variants showed hydrogel formation and therefore fluorescence, a cell mixture containing 40% active variants could be enriched to 89% active variants in a single round of screening [45,50]. For further characterization, the 10% YmPh variants showing the highest fluorescence were collected and their activity was determined using a microtiter-based activity assay using 4-MUP. An increased activity was detected for 11 YmPh variants, of which the best variant showed a 31% increase in *k*_cat_ towards 4-MUP compared to the wild type. In addition, this mutant also showed a 5% increase in *k*_cat_ for the natural substrate phytic acid in comparison with wild type YmPh [45].

Recently, Lülsdorf *et al.* showed that the fur-shell technology is also applicable to other hydrolases. They used *Bacillus licheniformis* *p*-nitrobenzyl esterase (pNBEBL), *Bacillus subtilis* lipase A (BSLA) and a cellulase from a metagenome library (CelA2). pNBEBL and BSLA hydrolyzed β-d-(+)-glucose pentaacetate, whereas CelA2 converted the β-d-cellobioside 4-MUC. After a single round of directed evolution, the *k*_cat_ of the best pNBEBL mutant towards β-d-(+)-glucose pentaacetate was increased seven-fold compared to the wild type. A BSLA variant was identified showing a 1.3-fold increased *k*_cat_ towards the screening substrate *p*-nitrophenyl acetate (*p*NPA) compared to the wild type. Finally, the catalytic activity of CelA2 was improved as well: the *k*_cat_ towards the β-d-cellobioside (4-MUC) substrate was enhanced by 1.9-fold compared to the wild type [50].

Cellular high-throughput encapsulation solubilization and screening (CHESS) was originally developed to increase the thermostability and detergent stability of G-protein coupled receptors (GPCRs) to facilitate crystallization. Single *E. coli* DH5α cells expressing the protein of interest are encapsulated by layers of the oppositely charged polymers chitosan and alginate [47,51]. The cell membrane is solubilized leaving only the capsule as a semipermeable barrier between cell components and the environment. The main advantage of the barrier is that small molecules such as fluorescent substrates and ligands can diffuse into the capsules whereas large proteins and the genetic information are retained: the molecular weight cut-off is 70 kDa. Therefore, a protein of interest smaller than 70 kDa needs to be expressed as a fusion protein, e.g., partnering with GFP or MBP molecules. Importantly, CHESS capsules are only stable in the range of pH 6–9: below pH 6 the capsules will aggregate, above pH 9 the capsules will dissolve [52].

CHESS was first applied to the rat neurotensin receptor (NTS_1_); the randomized mutant library StEPM303 [53] expressed in *E. coli* DH5α cells was encapsulated with alginate and chitosan, and subjected to a mixture of detergents. Next, the capsules were incubated with the fluorescent ligand BODIPY-FL-labeled neurotensin 8–13 (FL-NT), after which they were screened for expression of the membrane protein using FACS. In each of the three screening rounds, the top 0.5%–1% of the fluorescent capsules were collected. Finally, DNA from 20,000 collected capsules was recovered and analyzed for the ability of selected variants to bind FL-NT in *n*-heptyl-β-d-thioglucopyranoside (HTG). The best mutant NTS_1_-H4 showed an increase in thermostability of 21.6 °C for the bound and 26.8 °C for the apo state compared to the wild-type NTS_1_ [54].

CHESS has also been applied to soluble proteins, shown by optimization of the detergent stability and thermostability of superfolder green fluorescent protein (sfGFP). An epPCR library of 1.2 million sfGFP mutants fused to MBP was expressed in *E. coli* DH5α and encapsulated in chitosan and alginate. Before FACS screening the capsules were heated at 60 °C. The 1% capsules showing the highest fluorescence were collected, and DNA extracted from these capsules was subjected to two more rounds of screening with increasing temperatures for each round. Two mutants were identified: the very stable GFP (vsGFP) showed an increase in melting temperature of 12.9 °C in 2% (*w*/*v*) SDS over the wild type, while the ultra-stable GFP (usGFP) exhibited even a 19.3 °C improvement in 2% (*w*/*v*) SDS [52].

The gel-shell bead (GSB) approach also makes use of LbL technology for compartmentalization [48]. For the generation of GSBs, single *E. coli* BL21(DE3) cells expressing the protein of interest are compartmentalized in water-in-oil emulsion droplets using a microfluidics droplet generator. One aqueous stream contains the cells, liquid agarose and alginate; the aqueous stream is then mixed with a cell lysis reagent and a fluorescent substrate for catalysis. The catalytic reaction can be precisely controlled and is stopped by heat inactivation of the enzyme followed by cooling to 4 °C to solidify the agarose, thus forming a gel bead. Since gel beads have a ~250 kDa cut-off, an additional layer or shell is needed to maintain a link between genotype and phenotype. The additional shell around the gel beads reduces the molecular weight cut-off to ≤2 kDa and therefore guarantees the retention of DNA, protein and fluorescent product within the gel-shell beads. After sorting by FACS, genes of improved variants can be recovered by increasing the pH to 12 to disrupt the beads [48]. GSBs can also be recovered after use, offering the advantages of immobilized enzymes, though without having to express, purify and immobilize the enzymes. In comparison to the CHESS capsules described previously, GSBs are not only stable at high temperatures (up to 95 °C) and in the presence of organic solvents, but at pH values between 2 and 11 as well. However, microfluidic devices are needed for the generation of GSBs whereas CHESS capsules can easily be formed using standard laboratory equipment.

GSBs were first applied in the improvement of a phosphotriesterase (PTE) from *Pseudomonas diminuta* towards its native substrate, the pesticide paraoxon. Fischlechner *et al.* created an epPCR library of 5 × 10^5^ clones. After a single round of screening followed by an analysis in a 96-well plate for the turnover of the pesticide paraoxon, the best mutant PTE^F9^ was selected: it exhibited an eight-fold improved *k*_cat_/*K*_M_ for the pesticide paraoxon compared to the parent PTE^R8^ [48].

**Table 1:** FACS-based screening platforms described in this review.

Protein	Experiment	Compartment	Result	Throughput (Events/s)
**Nature’s own compartments**				
Antibodies (IgG) [14]	Selection and affinity maturation	Whole cells, mammalian display	Improvement in binding affinity towards human cytokine (hβNGF)	ND
*Staphylococcus aureus* sortase A (SrtA) [20]	Mutagenesis (epPCR, DNA shuffling and saturation mutagenesis)	Whole cells, yeast display	140-fold improvement in LPETG-coupling activity	ND
*S. aureus* SrtA mutant (eSrtA [20]) [22]	Mutagenesis (epPCR, DNA shuffling and saturation mutagenesis)	Whole cells, yeast display	51,000-fold change in specificity for LAETG instead of LPETG and a 125-fold change in specificity for LPESG instead of LPETG	ND
Tobacco Etch Virus protease (TEVp) [24]	Mutagenesis via epPCR	Whole cells, yeast display	1100–5000-fold reversed substrate specificity	~2 × 10^8^ cells screened
TEVp [26]	Enrichment	Whole cells, intracellular expression	69,000-fold enrichment for variants recognizing the natural substrate	300
TEVp [30]	Site-directed mutagenesis	Whole cells, intracellular expression	Substrate profiling	300
*Pseudomonas plecoglossicida* arginine deiminase (PpADI) [31]	Mutagenesis (epPCR)	Whole cells, intracellular expression	2.8-fold increase in *k*_cat_/*K*_M_ in comparison to the parent PpADI mutant M21	5000
**Man-made compartments (emulsion-based)**				
Cellulase Cel5A [12]	Enrichment	Whole cells, double emulsion droplets	12-fold enrichment of active variants form a mixture containing 5% cells expressing cellulase	8000 to 20,000
Subtilisin Carlsberg (SC) [36]	Mutagenesis (epPCR)	Whole cells, double emulsion droplets	160% increase in resistance towards antipain dihydrochloride	8000
α-Hemolysin [37]	Mutagenesis (epPCR)	Cell-free, liposome display	30-fold higher pore-forming activity	ND
**Man-made compartments (polymer-based)**				
*Yersinia mollaretii* phytase (YmPh) [45]	Mutagenesis (epPCR)	Whole cells, fur-shell	97 U·mg^−1^ higher specific activity towards 4-methylumbelliferylphosphate (4-MUP)	5000
*p*-nitrobenzyl esterase (*Bacillus licheniformis*); lipase A (*Bacillus subtilis*); cellulase (CelA2) (metagenome library [55]) [50]	Mutagenesis (epPCR)	Whole cells, fur-shell	7-fold increase in *k*_cat_ towards *p*NPA; 1.3-fold increase in *k*_cat_ towards β-d-(+)-glucose pentaacetate; 1.9-fold increase in *k*_cat_ towards 4-methylumbelliferyl-β-d-cellobioside, respectively	5000
G-protein coupled receptors (GPCRs) [54]	Mutagenesis (StEP, Slonomics^®^ technology [53], Martinsried, Germany)	Whole cells, CHESS	~26.8 °C increase in thermostability of NTS_1_	8000
sfGFP [52]	Mutagensis (epPCR)	Whole cells, CHESS	~19 °C increase in thermostability in 2% (*w*/*v*) SDS	8000
*Pseudomonas diminuta* phosphotriesterase [48]	Mutagenesis (epPCR)	Cell lysate, GSBs	19-fold increase in *k*_cat_/*K*_M_ towards tetraethyl-*O*-fluorescein diphosphate	~2800

ND indicates the throughput has not been determined or stated; epPCR, error-prone polymerase chain reaction; CHESS, cellular high-throughput encapsulation solubilization and screening; GSB, gel-shell beads.

## 4. Microfluidics Screening Platforms

Despite a significant extension of the versatility of conventional FACS by application of different compartmentalization systems, FACS-based screening platforms still face some limitations. The main problem is that the compartments cannot be formed and manipulated (e.g., droplet fusion, fission, or mixing) within the experiment in a controlled manner [56]. This leads to the formation of highly polydisperse compartments or to polymer aggregation. Consequently, compartments of different sizes carrying proteins with the same activity can lead to incomparable assay outcomes [57,58]. In addition, bulk emulsion droplets are hard to manipulate: it is very difficult and sometimes even impossible to add new reagents to pre-formed droplets at defined times [9,59].

**Figure 6:**
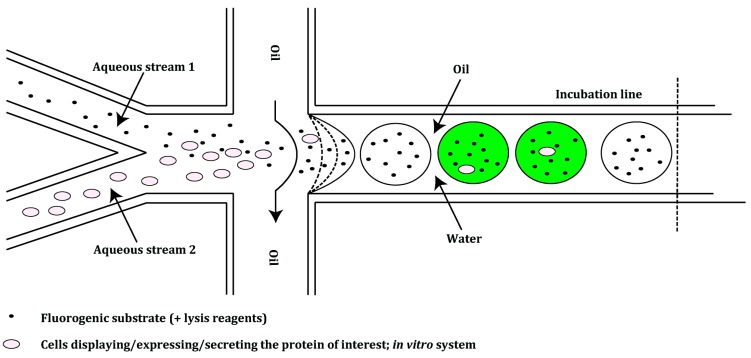
Representation of a droplet generator module of a microfluidic screening platform. Aqueous stream 1 contains the fluorogenic substrate which can be supplemented with lysis reagents. The stream is combined with aqueous stream 2, which carries a suspension of cells displaying, expressing or secreting the protein of interest and variants thereof, or a PCR mixture (*in vitro* systems). The water-in-oil droplets are formed at a flow-focusing junction and move into an incubation line. Droplets carrying active variants are shown in green. The incubation line is either directly connected to the next module of the platform (sorting module, Figure 6) or it is intersected with an additional module—the droplet fusion device (not depicted)—which enables modification of preformed droplets [59,64,65,68,69,70].

**Figure 7:**
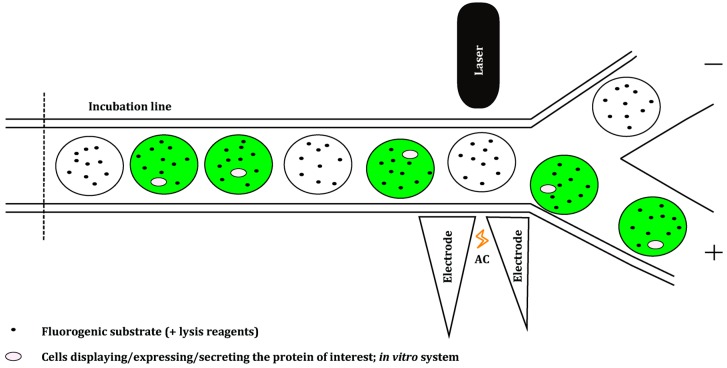
Representation of a droplet sorting module of a microfluidic screening platform. The module consists of a sorting junction controlled by a fluorescence detector and electric field applied across two electrodes. Each of the generated emulsion-based droplets is examined with respect to its fluorescence. The presence of fluorescence (green droplets) activates the alternating current (AC) electrodes, which results in the formation of the electric field. Once droplets come into contact with the electric field, the dielectrophoretic activity of the droplets is triggered: they will become polarized. Consequently, the polarized droplets can move in two directions at the junction. If the dielectric constant of a droplet is higher than the conductivity of the electric current, the droplets will move in the direction of the increasing electric field (+). In contrast, if the dielectric constant of a droplet is lower, the droplets move away from high field regions (−) [59,64,65,68,69,70].

To overcome the limitations mentioned above and to increase the throughput of screening platforms, new, faster and more economical approaches are being investigated. Microfluidics enable droplet manipulation, incubation and sorting all in one system [60]. Different droplet-based microfluidic modules have been developed, which can control the formation of highly monodisperse single [61] and double emulsions [62] (Figure 6). Other modules can measure reaction kinetics in microfluidic systems [63] and sort droplets [64] (Figure 7). Additionally, methods for the delivery of fluorogenic substrates into the droplets have emerged; adding a substrate into the stream carrying an enzyme is usually done prior to microdroplet formation [65,66]. Fluorogenic substrates can also be injected or fused with a pre-formed droplet at a defined time, depending on the kinetics of the enzymatic reaction. This enables adjustment of the reaction’s incubation time prior to selection [59,67,68].

Nevertheless, microfluidic platforms pose a technical challenge [57]. To build microfluidic devices, specialized and non-standard equipment is needed. Therefore, FACS-based screening methodologies using standard equipment are still favored. However, commercial microfluidic devices, in particular microfluidics-based modules for droplet formation, are becoming more easily available [71]. These modules can be used for the formation of single and double emulsions [72], and GSBs [48]. As double emulsions and GSBs are compatible with standard cell sorters, a pipeline can be created from the creation of capsules using microfluidic devices to the analysis; this will enable high-throughput screening of libraries for directed evolution [59,68]. An overview of screening platforms based on compartmentalization by microfluidics is given in Table 2.

The first successful microfluidic-based screening application in directed enzyme evolution was designed for the selection of improved catalysis of horseradish peroxidase (HRP) towards Amplex Ultrared (AUR) [65]. In this method, an HRP mutant library is displayed on the yeast cell surface. The yeast cells enter the first microfluidics module via one stream, where they are combined with a second stream carrying the fluorogenic substrate AUR. Upon mixing of these two streams, picoliter-volume aqueous drops are formed at a flow-focusing junction in fluorocarbon oil. Next, the drops are pooled in an incubation line, which is connected to the screening module. There, the droplets are dielectrophoretically sorted according to the fluorescence signal of the reaction product, which is retained within the droplet.

Two generations of horseradish peroxidase variants were analyzed. In the first generation, two types of libraries were created by epPCR and site-specific mutagenesis, each containing >10^7^ mutants of the HRP gene. Four sorting rounds of the first generation of both libraries gave a ~2 times increase in the mutants’ catalytic efficiency towards the substrate AUR. The 18 best variants were further randomized to create a second generation of the HRP mutants. From a pool of 10^7^ variants, the 5%–10% droplets with the highest fluorescence were sorted; the 13 best mutants after five rounds of screening all showed a significant improvement in *k*_cat_/*K*_M_ with the best mutant showing a seven-fold increase over the wild type HRP. In comparison to robotic microtiter plate screening methods, the microfluidic droplet-based approach proved to be 1000-fold faster in the screening of a mutant library [65].

Hollfelder and co-workers extended the microfluidics system described above to a bacterial cytoplasmic expression system at a picoliter scale [66]. In the first microfluidic device, an aqueous suspension of bacterial cells expressing the enzyme of interest is mixed together with a second aqueous stream at a flow-focusing junction. The second stream not only carries a substrate for PAS, but is also supplemented with cell lysis reagents. After mixing of both streams followed by droplet formation, *E. coli* cells are lysed and protein variants are released into the droplet; during off-chip incubation, active enzyme variants convert the substrate into a fluorescent product. Then, droplets are injected into the second microfluidic device, where they are sorted dielectrophoretically based on their fluorescence signal.

Directed evolution was applied to increase the promiscuous hydrolytic activity of the arylsulfatase from *Pseudomonas aeruginosa* (PAS) towards the non-native substrate phosphonate. A PAS mutant library was expressed in the cytoplasm of *E. coli* BL21(DE3) cells. After three rounds of sorting in which the top 4% brightest droplets were collected, 500 mutants were further analyzed for their promiscuous activity. The best mutant showed a six-fold improved hydrolytic activity (*k*_cat_/*K*_M_) towards the non‑native substrate phosphonate compared to the wild type PAS. This method proved to be a 100 times faster than advanced robotic microtiter plate-based screening systems [66].

In another approach proposed by Romero *et al.* the characterization of a library of enzyme variants was done by means of fluorescence-based activity assay followed by deep mutational scanning [71]. In this method single *E. coli* BL21(DE3) cells expressing a library of enzyme variants are combined with a substrate and the lysis reagents in the microdroplets using a microfluidic co-flow droplet generator. After an off-line incubation, droplets are injected into a microfluidic sorter, where they are analyzed based on the level of the fluorescence. Selective electrocoalescence is used to separate 3.4% of positive drops that exceeded the threshold of 0.2 fluorescence [73]. Next-generation sequencing data obtained from a sorted and an unsorted library are used for the statistical analysis of the frequency of mutations that are found in analyzed variants [74]. Deep mutational scanning is a very powerful and promising approach that can help to explore the protein fitness landscape by answering fundamental questions on the dependence of the protein’s function on mutations [75]. This approach was used to map the activity of 10 million epPCR variants of β-glucosidase (Bgl3) from *Streptomyces* sp. [71]. In this method, *E. coli* BL21(DE3) cells expressing the library were analyzed for their activity towards the substrate fluorescein di-(β-d-glucopyranoside). The droplets were collected and incubated off-chip, after which the droplets were injected into the microfluidic sorting device where they were screened for fluorescence emission. Prior to sorting, 35% of the library members were found to be functional, whereas, after sorting, the fraction of functional variants had increased to 98%. Genes recovered from the active droplets were subjected to sequencing analysis to enable high-throughput sequence-function mapping. As a result of statistical analyses, a large decrease in the mutation frequency was observed for the sorted library when compared with the results obtained for the unsorted library carrying active and inactive variants. This suggests that mutations that were not found in the sorted library were unfavorable for the proper function of the enzyme. The less tolerant the residue was to mutation, the more that position denoted its functional importance.

Furthermore, to demonstrate the versatility of this platform, the same screening experiment was conducted under altered conditions: droplets carrying Bgl3 variants were subjected to a 10-min heat challenge at 65 °C to improve the enzyme’s thermostability. After a sorting step, 20% of the initial population still showed enzymatic activity. The highest impact on the enzyme’s thermostability reached a 5.3 °C higher tolerance [71].

The microfluidics platforms described so far make use of bacterial or yeast cells as DNA library carriers. Cell-free systems overcome limitations associated with the use of *in vivo* expression systems [76]. A completely *in vitro* screening system has been developed and its applicability in protein engineering experiments has been demonstrated by means of an enrichment experiment for the enzyme β-galactosidase [68]. Here, the *lac*Z gene and *lac*Z*mut* genes encoding inactive enzyme variants were amplified by PCR and mixed into monodisperse droplets. These droplets were fused with droplets carrying purified *in vitro* transcription and translation (IVTT) components, supplemented with the fluorogenic substrate fluorescein di-(β-d-galactopyranoside) (FDG). During a 2 h off-chip incubation, the *lac*Z and the *lac*Z*mut* genes were transcribed and translated, and droplets carrying the active β-galactosidase converted the substrate FDG to galactose and fluorescein. After injection into the sorting device, the droplets were sorted by fluorescence-activated electrocoalescence (2000 droplets/s), allowing for a direct recovery of DNA from the aqueous stream [68]. As a result, *lac*Z genes encoding an active β-galactosidase could be enriched 502-fold from a 1:100 molar ratio mixture of *lac*Z to *lac*Z*mut* genes. This droplet-based system enabled a reduction of the volume of reagents by ~78,000-fold and an increase in sorting speed up to 2000 droplets/s. Consequently, this system turned out to be 100,000-fold more economical when compared to microtiter plate‑based systems. With a still 10-fold lower sorting rate, the described microfluidic system compensates for this by being more quantitative and flexible than FACS methods based on double emulsions.

Recently, microfluidic devices were used for the enhancement of production hosts of industrial enzymes: a yeast cell library was screened for an improved homologous production of secreted α-amylase [69]. Yeast strain MH34 was subjected to irradiation with UV light, thus randomly introducing mutations throughout the whole genome. Next, each cell was encapsulated in emulsion droplets together with the substrate starch conjugated with the internally quenched BODIPY fluorophore. During the 3 h off-chip incubation, the enzyme was produced and secreted into the emulsion droplet. While the starch substrate was converted, the BODIPY fluorophores became gradually unquenched. The generated emulsion containing converted substrate was injected into a separate sorter circuit. Each droplet was screened and sorted based on its fluorescence value. Only the droplets that exceeded the threshold value of the top 0.72% most fluorescent population were qualified as active and collected. Cells from the sorted population showed on average a 63% higher enzyme production and a 35% higher product yield compared to the unsorted library. Sixty individual clones were selected for further characterization. In addition to a higher growth rate, the best clone showed twice the α-amylase production of the mother strain. The application of a droplet-based microfluidic system instead of a commonly used automated microtiter plate screening system enabled the analysis of about 3 × 10^6^ droplets at a rate of 323 droplets/s, an over 300 times higher throughput. This method could have a significant industrial value, not only because of the higher rate of library screening but also because of the million-fold reduction of reagent consumption [69].

The application of a droplet-based microfluidics platform for metagenomic studies was demonstrated by the enrichment of cellulolytic microorganisms from environmental samples [77]. Cellulases are a mixture of endoglucanases and exoglucanases or cellobiohydrolases working synergistically together; they can efficiently hydrolyze cellulosic biomass into fermentable sugars, which are used for the production of valuable biofuel. Najah *et al.* screened a soil sample from a wheat stubble field for cellobiohydrolase activity. Bacteria were directly extracted from the soil sample and compartmentalized in water-in-oil droplets using a microfluidic drop-maker device. Each droplet contained a fluorogenic cellobiohydrolase substrate, β-d-cellobioside-6,8-difluoro-7-hydroxycoumarin-4-methanesulfonate [77]. The generated emulsions were incubated off-chip for 24 h at 30 °C. Next, they were injected into a fluorescence-activated droplet sorting device. The enzymatic activity in each droplet was assessed by the fluorescence obtained from the product of the hydrolysis reaction, sorting the 2% droplets with the highest fluorescence. The cellulase activity of the selected bacterial population was also assessed using a microtiter plate growth assay containing sodium β-d-cellobioside-6,8-difluoro-7-hydroxycoumarin-4-methanesulfonate. Both methods enabled the discovery of a diverse population of cellulolytic bacteria directly from soil. Nevertheless, the droplet-based method resulted in the selection of a higher cellobiohydrolase activity compared to the plate growth assay: this population exhibited a 17-fold higher exoglucanase activity as well as a seven-fold endogluconase activity, the latter being important in the collective reaction of cellulose hydrolysis. A metagenomics screen revealed a difference in the taxonomic diversity obtained after screening: after the microfluidic screening, *Paenibacillaceae* and *Paenibacillus* species were most abundant, whereas after the microtiter-based method, the *Bacillaceae* species was predominantly found. All in all, the use of the droplet-based microfluidic system enabled the screening of 100,000 bacteria in less than 20 min. The use of a state-of-the-art robotic microtiter plate screening system would still require more than three days to screen the same number of microorganisms. In addition, the reagent volume and costs were ca. 250,000-fold lower for the microfluidic system [78,79].

**Table 2:** Microfluidics-based screening platforms described in this review.

Protein	Experiment	Compartment	Result	Throughput (Events/s)
Horse radish peroxidase [65]	Mutagenesis (epPCR and saturation mutagenesis)	Yeast display, drop-based microfluidic system	7-fold increase in catalytic efficiency towards Amplex Ultrared (AUR)	2000
*Pseudomonas aeruginosa* arylsulfatase [66]	Mutagenesis (epPCR)	Cell lysate, drop-based microfluidic system	6-fold increase in promiscuous hydrolytic activity towards the nonnative substrate phosphonate	926
*Streptomyces* sp. β-glucosidase [71]	Mutagenesis (epPCR)	Cell lysate, drop-based microfluidic system combined with high-throughput DNA sequencing	5.3 °C increase in thermostability	>100
β-galactosidase [68]	Enrichment	*In vitro* transcription and translation, drop-based microfluidic system	502-fold enrichment of positive variants from a mixture of active and inactive variants	2000
Yeast strain MH34α-amylase [69]	Mutagenesis (UV irradiation)	Whole cells, drop-based microfluidic system	2-fold increase in α-amylase production	323
Cellulases for the hydrolysis of cellulosic biomass [79]	Metagenomics	Whole cells, drop-based microfluidic system	Identification of microorganisms with 17-fold higher cellobiohydrolase activity and 7-fold higher endogluconase activity	6667

## 5. Future Perspectives

The continuous evolution of protein engineering strategies is leading to a faster and more specific development of valuable (therapeutic) proteins and, in particular, enzymes [80,81]. Apart from directed evolution methods used in the techniques described in this review, it is important to mention that rational approaches are also increasing in power [82,83]. No matter which method is used or how smart the library is, the identification of desirable variants can still be challenging and time-consuming. Thus, there is an obvious need for flexible and fast screening methods. A general direction observed in the strategies for library screening is the miniaturization of reaction volumes. As such, screening technologies are taken to the level of natural compartments—typical bacterial cells have volumes of a few femtoliters [9].

Microencapsulation approaches combined with FACS analysis have been broadly exploited for protein engineering [9]. Nevertheless, only a few enzymes were reported to be routinely engineered using the water-in-oil or double emulsion methods [4,9]. One of the main technical difficulties of these techniques has been the generation of monodisperse droplets [4]. New technologies based on polymerization reactions or the use of microfluidic devices have enabled generation of compartments in more controllable way [9,79].

Microfluidic systems provide the ability to screen an extensive number of compartmentalized reactions in one experimental set-up. They also allow monitoring the actual activity levels of single molecules or in single cells. By integrating droplet-maker and manipulation modules with sorting devices, it has become possible to perform sophisticated assays for directed evolution. Currently, screening platforms are based on the measurement of a fluorescent product. However, other methods for optical readouts will become available in the future; some work has already been done on the development of microfluidic systems which can use absorbance [84], Raman scattering [85], or ultrasound [86] detection as a means to determine enzymatic activity.

Finally, the drawbacks and advantages of FACS-based and microfluidics-based screening platforms are summarized in Table 3. At present, the most versatile as well as user-friendly high-throughput screening procedure in protein engineering seems to be a system comprising of both techniques combined [57]. The sorting speed of microfluidics-based platforms is still below the capabilities of fluorescence-activated cell sorters (FACS) (sorting rates up to 50 kHz; 2000 droplets/s). However, it is probably only a matter of time until droplet sorters are able to screen at a higher speed; a new design enabling sorting 10 times faster than the fastest available droplet sorter has already been reported. With >99% accuracy, it can sort ~10^8^ droplets/h at rates up to 30 kHz, and sorting can still be accelerated. Currently, the limiting factor is therefore not the physical mechanism of sorting but rather the electronics to detect the droplets [87]. Once these obstacles are removed, these microfluidics-based platforms will be of key importance in the directed evolution of enzymes and (therapeutic) proteins.

**Table 3:** Comparison of FACS-based and microfluidics-based screening platforms.

FACS-Based	Microfluidics-Based
The utility of FACS assays is limited to fluorophores that remain inside or on the surface of cells	The utility of microfluidics is broadened to components that are secreted
Water-in-oil emulsions must be converted into a water-in-oil-in-water emulsion (double emulsion)	Water-in-oil emulsions can be sorted directly
Pre-formed droplets are difficult to manipulate (restricted range of assays)	Pre-formed droplets are easy to manipulate: they can be divided, fused, incubated, analyzed, sorted, broken up
Limited control over the reaction conditions in a droplet	Much greater control over the reaction conditions in a droplet
Lack of control over the droplet volume, leading to polydispersity	Good control over the droplet volume, highly monodisperse
Requires standard cell sorters	Requires specialized instrumentation
Sorting speed up to 20,000 droplets/s	Sorting speed up to 2000 droplets/s

FACS—fluorescence-assisted cell sorting.
